# Maternal and newborn health inequality among Syrian refugees in Turkey: a systematic review and meta-analysis

**DOI:** 10.1186/s12939-025-02506-2

**Published:** 2025-06-02

**Authors:** Sevil Hakimi, Esin Ceber Turfan, Leila Allahqoli, Mohadeseh Ahmadi, Neriman Sogukpinar, Mahide Demirelöz Akyüz, Esmat Mehrabi, Azam Rahmani, Ibrahim Alkatout

**Affiliations:** 1 Faculty of Health Sciences, EGE University, Izmir, Turkey; 2 Ministry of Health and Medical Education, Tehran, Iran; 3 Faculty of Medicine, School of Population and Public Health, University of British Columbia, Vancouver, Canada; 4 Faculty of Nursing and Midwifery, Tabriz University of Medical Sciences, Tabriz, Iran; 5 Nursing and Midwifery Care Research Center, School of Nursing and Midwifery, Tehran University of Medical Sciences, Tehran, Iran; 6 Department of Gynecology and Obstetrics, Kiel School of Gyanecological Endoscopy, Arnold-Heller-Str. 3, Haus C. 21405, Kiel, Germany

**Keywords:** Health inequalities, Maternal health, Newborn health, Refugees

## Abstract

**Objective:**

In this meta-analysis we explore significant health disparities in maternal and newborn health among Syrian refugees residing in Turkey.

**Method:**

The study protocol was registered in PROSPERO. We conducted a comprehensive literature search across six databases, including sources in English and Turkish, as well as relevant UN agencies, covering the period from 2011 (the onset of the Syrian conflict) to September 2024. This research specifically targets Syrian mothers aged 15 to 49 who were either pregnant or had recently given birth in Turkey, including studies with observational cross-sectional or retrospective designs. The quality of the included studies was evaluated using the JBI Critical Appraisal Checklist. Statistical analyses were performed using R version 4.4.1.

**Result:**

Of 382 studies in English and Turkish, 29 papers, 2 reports and 1 postgraduate thesis were selected for full-text evaluation. Syrian migrants were more at risk of anemia in the third trimester of pregnancy [RR: 2.27 (95% CI: 1.57 to 3.32)], and had less access to antenatal care [RR: 0.39 (95% CI: 0.26 to 0.58)] and iron supplementation during pregnancy [RR: 0.69 (95% CI: 0.46 to 0.96)] compared to the native population. The risks of adolescent pregnancy [RR: 3.78 (95% CI: (3.06 to 4.88)] and home birth [RR: 3.68 (95% CI: (2.53 to 5.27)] were higher among migrants [RR: 3.78 (95% CI: (3.06to 4.88)]. Conversely, migration was an important factor in gestational diabetes [RR: 0.44 (95% CI: (0.21 to 0.90)] and newborn macrosomia [RR: 0.54 (95% CI: (0.50 to 0.58)] as well as preeclampsia [RR: 0.56 (95% CI: (0.32 to 0.98)].

**Conclusion:**

Our data revealed that Syrian migrant mothers face a higher risk of anemia, limited access to antenatal care and iron supplements, and higher rates of adolescent pregnancies and home births compared to their native counterparts. However, migration appears to have a protective effect on gestational diabetes and preeclampsia. The results underscore the need for targeted health interventions and policies that address access to maternal healthcare services.

## Introduction

Health inequalities strain healthcare systems, reinforce social injustice, and hinder societal development [1, 2]. Rooted in social, economic, and environmental determinants, these disparities extend beyond individual health outcomes, threatening social cohesion and economic stability [3–6]. Refugee populations are particularly vulnerable due to complex barriers in host countries, including language challenges, cultural differences, poor living conditions, and systemic unfamiliarity with healthcare services [7–9].

Women and children among refugee communities face disproportionate health risks such as mental health disorders, preterm birth, low birth weight, and increased maternal and neonatal mortality [10, 11]. The perinatal period represents a high-risk phase for refugee mothers, as inadequate access to comprehensive and culturally appropriate healthcare often results in delayed engagement with health professionals and insufficient support systems [10–13]. These challenges are amplified by the intersection of refugee status, gender, and maternal health, necessitating targeted and culturally sensitive healthcare interventions [14].

Since the onset of the Syrian conflict in 2011, Turkey has become the largest host country for Syrian refugees. As of August 2024, approximately 3.5 million Syrian refugees reside in Turkey. By the end of 2023, children under 4 years made up 14.5%, while women aged 15–49 constituted nearly 23% of this population [15, 16]. Despite growing attention to refugee health issues [17–19], comprehensive data focusing on maternal and newborn health among Syrian refugees in Turkey remains scarce. This meta-analysis aims to address inequalities in maternal and newborn health outcomes between Syrian and Turkish mothers. By highlighting this population's unique vulnerabilities, the study supports the development of tailored, equity-driven public health strategies and provides a foundation for future research and policymaking aligned with global health goals.

## Methods

### Protocol and guideline

The study protocol was registered in advance in the International Prospective Register of Systematic Reviews (PROSPERO) and was given the ID CRD42024583624. The systematic review and meta-analysis followed the guidelines established by the Preferred Reporting Items for Systematic Reviews and Meta-Analyses (PRISMA) framework [20].

The research questions were developed using the PICO framework, focusing on health inequalities in maternal and newborn health among Syrian refugees living in Turkey. Population: The study population consisted of Syrian refugee mothers and their newborns. Intervention: There was no intervention involved in the study. Comparison: The comparison was made with Turkish mothers and newborns. Outcomes: Outcomes were maternal and newborn health indicators, which are detailed in the measurement section of the report.

### Search strategy and information sources

We conducted a comprehensive literature search on the following electronic databases: PubMed, Scopus, Web of Science, EMBASE and the Turkish Academic Network and Information Center (ULAKBIM), including journals accessed via DergiPark. Additionally, we carried out a manual search via Google Scholar, UN agency websites including the United Nations Fund for Population Activities (UNFPA) and the United Nations High Commissioner for Refugees (UNHCR), in order to obtain reports and conference papers related to the keywords. The search was limited to studies published in the English and the Turkish language between 2011 (the beginning of the Syrian conflict) and September 2024.

The search terms were developed as follows using a combination of keywords relating to the study population and outcome indicators: “Syrian women” OR “Syrian migrants” OR “Syrian migrant women” OR “Syrian refugees” OR “Syrian mothers” AND “health inequality” OR “inequalities”, AND"maternal"OR"pregnancy"OR “antenatal care” OR “child health”, AND"newborn"OR"newborn health".

### Inclusion and exclusion criteria

The investigation was focused on Syrian mothers aged 15–49 who were pregnant or had given birth in Turkey, and included studies with an observational cross sectional or retrospective design.

### Inclusion criteria

We included peer-reviewed English or Turkish articles and official reports that compared maternal and newborn health outcomes between Syrian migrants living in Turkey and the native Turkish population, as well as studies that focused specifically on Syrian mothers and newborns living in Turkey. These inclusion criteria ensured that we captured a wide range of studies addressing health outcomes among Syrian migrants in Turkey, whether in comparison to the native Turkish population or within the Syrian migrant community. Data were collected retrospectively from medical records or during labor and childbirth through questionnaires and interviews. When interviews were conducted, indicators such as gestational diabetes and preeclampsia/eclampsia were identified through medical reports. The family income status was assessed on the basis of the participants'perceptions and categorized into low, moderate, or high.

Women and newborns were divided into two distinct groups:

Syrian refugees: This group consisted of Syrian-born women who had fled their homes in Syria due to the civil war that began in 2011, and had given birth in Turkey.

Turkish population: This group consisted of women born and residing in Turkey, and served as controls. Notably, newborns of both groups were born in Turkey.

### Exclusion criteria

We excluded studies that did not specifically focus on Syrian migrants in Turkey, those that did not report on maternal and newborn health outcomes, qualitative research, systematic reviews and meta-analyses, scoping reviews, narrative reviews, editorials, and opinion pieces.

### Selection process

**Fig. 1:**
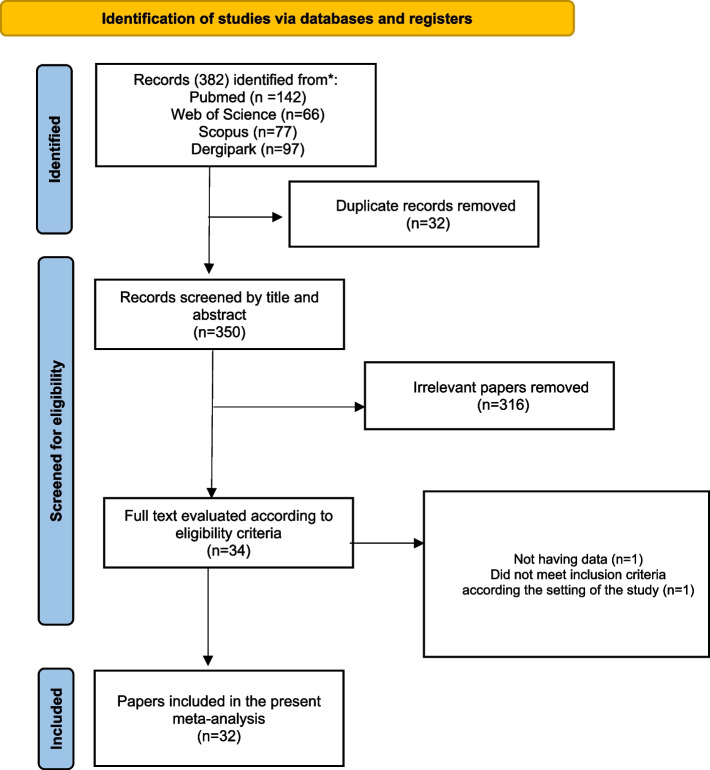
PRISMA flow chart

The EndNote software (version X9, Thomson Reuters), which aided in listing and screening studies, was used for the selection process. The latter was divided into three distinct phases: screening, selection, and data abstraction. During the screening phase, two trained authors (LA and AR) independently evaluated the titles and abstracts of the identified studies. In the selection phase, two authors (EC and NS) independently assessed the full-text articles against the inclusion criteria using a checklist-style form. Articles meeting the inclusion criteria were incorporated in the final analysis. To ensure consistency and resolve any discrepancies, a third expert (SH) reviewed the full-text articles and addressed any inconsistencies or disagreements that arose during the selection process. The selection process was displayed on a PRISMA flowchart (Fig. 1) showing how studies were screened and incorporated in the review.

### Outcomes

We considered several maternal and neonatal outcomes in the meta-analysis.

### Maternal health outcomes

- Adolescent pregnancy (age of mother ≤ 19 years, binary variable expressed as a percentage.) [21].
- Contraception method (use of oral contraception, condoms, intrauterine device, injection methods, binary variable expressed as a percentage.).
- Gestational diabetes according to medical records (binary variable expressed as a percentage.). GDM was diagnosed when one or more of the following plasma glucose values were met or exceeded during a 75 g oral glucose tolerance test (OGTT): fasting ≥ 92 mg/dL, 1-h ≥ 180 mg/dL, or 2-h ≥ 153 mg/dL [22].
- Pregnancy-induced hypertension/preeclampsia/eclampsia (binary variable expressed as a percentage) according to the medical records of participants: Blood pressure 140/90 mm Hg or higher, measured on two occasions at least four hours apart after 20 weeks of gestation; the presence of 300 mg or more of protein in a 24-h urine collection, or a protein-to-creatinine ratio of 0.3 or higher, or 1 + or greater on a dipstick test (if no other tests were available) [23].
- Mode of delivery (vaginal or cesarean section, binary variable expressed as a percentage.).
- Maternal anemia according to the medical records of the participants (Hb ≤ 11 g/dl at the end of pregnancy or during the third trimester, binary variable expressed as a percentage.) [24].

### Newborn health outcomes

- Preterm birth according to the medical records of the participants (birth before 37 weeks of gestation, binary variable expressed as a percentage.) [25].
- Congenital malformations, binary variable expressed as a percentage.
- Stillbirth, binary variable expressed as a percentage.
- Admission to the neonatal intensive care unit according to the medical records (NICU), binary variable expressed as a percentage.
- Low birth weight according to the medical records (birth weight < 2.5 kg for term infants, binary variable expressed as a percentage.) [26].
- Macrosomia according to the medical records (birth weight ≥ 4 kg for term infants, binary variable expressed as a percentage.) [27].

### Data extraction

Study data were initially extracted by two researchers (MA and AR) using a predefined Excel form. To ensure accuracy, the extracted data were cross-checked by two other researchers (MDA and EM).

The data extraction form included several essential elements such as the authors’ names, year of publication, study design, sample size, target population, place of data collection, the participants‵ income, educational status, and the reported prevalence of selected indicators.

### Quality assessment

The quality of the selected papers was evaluated using the JBI Critical Appraisal Checklist for Systematic Reviews and Research Syntheses. This checklist, designed for cross-sectional studies, comprises eight questions that emphasize methodological rigor, clarity in reporting, and the appropriateness of conclusions and recommendations. Reviewers may respond with a"yes"if the review clearly satisfies the criteria, or"no,""unclear,"or"not applicable"if it does not. Each criterion achieved is given a score of 1, with a maximum possible score of 8. Reviews scoring above 70% are classified as high quality, scores between 50 and 70% are considered moderate, and scores below 50% are categorized as low quality [28].

### Statistical analysis

Statistical analysis was performed using R version 4.4.1. The characteristics of participants were compared between the two groups. Quantitative variables such as age and pregnancies were analyzed using weighted means and standard deviations (SD) with the metacont function. Qualitative variables, including the prevalence of first-time mothers, low-income participants, and women with at least a high school diploma, were assessed using the metabin function to calculate risk ratios. Since all the health outcomes we compared in this study, were binary risk ration is an appropriate measure to express the strength of the association between exposures and these outcomes. So in the meta-analysis, maternal and newborn health outcomes of Syrian women (cases) were compared to those of Turkish women (controls) using risk ratios (RR). Data were extracted from various studies to evaluate the association between migration and health inequalities. An RR > 1 suggested a higher risk of the outcome, while an RR < 1 indicated a lower risk. The pooled estimates and 95% confidence intervals (CI) for each outcome were calculated. A random-effects model was employed to account for heterogeneity in study designs, populations, and outcome measures. Cochran's χ^2^ test assessed heterogeneity, with the I^2^ statistic indicating variability across studies. Statistical significance was set at a *p*-value less than 0.05. Forest plots were generated only for outcomes showing significant differences between migrants and the native population.

### Sensitivity analyses

Sensitivity analyses were conducted separately for maternal and child health outcomes. These included studies that lacked a comparator group and focused exclusively on migrant populations.

## Results

### Overview

A comprehensive literature search yielded 382 studies in English and Turkish, of which 32 duplicates in English were removed. After screening the titles and abstracts of the remaining 350 studies, we selected 31 papers, 2 reports and 1 post graduate thesis for full-text evaluation based on the inclusion criteria. After further evaluation, 2 studies were excluded because they failed to meet the criteria: 1 was conducted in Syria [29] and 1 was an editorial article without empirical data [30]. Ultimately, 29 papers, 2 reports and 1 thesis remained for analysis, as shown in the PRISMA flow diagram (Fig. 1).

Of the 32 included reports, 24 were written in the English language [31–55] and 8 in Turkish [56–60]. Two reports were issued by WHO Europe [61] and Hacettepe University's Institute of Population Studies [62], while one was a Master's thesis written in Turkish [63]. In terms of geographic location 6 studies were conducted in Istanbul, 4 in Ankara, and 2 reports presented results from nation-wide investigations. One report did not specify the exact location (Table 1). Women of Turkish origin comprised nearly 86.5% (*n* = 281,217) of the study population, while women of migrant origin accounted for 13.5% (*n* = 44,049). Of the 32 reviewed studies, 6 lacked comparator groups [32, 34, 47, 53, 64, 65] and were therefore included in the sensitivity analysis.

**Table 1:** Characteristics of eligible studies

NO	Study, year	Study design	Study population	Total sample size	Migrant sample size	Native sample size	Comparison group	Location of study	Language of paper
1	Saylan, 2024 [31]	Retrospective, using secondary data^1^	Women of reproductive age	4713	1958	2755	Native population	Rural, urban, and refugee camps	English
2	Dirican, 2024 [32]	Retrospective, using hospital data	Syrian women who had given birth between 2016 and 2020	2866	2866	NA	NA	Konya	English
3	Dogan, 2023 [33]	Cross-sectional, questionnaire	Pregnant women	290	290	NA	NA	Erciyes	English
4	Dogan, 2023 [34]	Cross-sectional, questionnaire	Pregnant women	400	200	200	Native population	Not identified Central Anatolia	English
5	Inal, 2023 [35]	Retrospective. hospital data	Syrian and Turkish women who had given birth between 2016 and 2020	17,997	3579	14,418	Yes	Konya	English
6	Çelik, 2022 [56]	Cross-sectional, questionnaire	Women married below the age of 18 years	109	52	57	Native population	Urban, Kilis	Turkish
7	Karaca Kurtulmus, 2021 [36]	Retrospective, hospital records	Women who gave birth	152,478	11,036	141,442	Native population	Izmir, Aydin	English
8	Sayili, 2021 [37]	Prospective cohort, hospital records	Pregnant women with high-risk pregnancies	302	69	233	Native population	Sanliurfa	English
9	Vural, 2021 [38]	Retrospective, hospital data	Syrian and Turkish women who had given birth between 2013 and 2018	55,254	8103	47,151	Native population	Izmir	English
10	Kanawati, 2021 [39]	Retrospective, hospital data	Syrian and Turkish women who had given birth between 2014 and 2016	1020	509	511	Native population	Istanbul	English
12	Turkay, 2020 [52]	Retrospective hospital data	Syrian and Turkish women who had given birth between 2016 and 2017	8570	620	7950	Native population	Kocaeli	English
13	Okman, 2020 [57]	Retrospective, hospital records	Women who gave birth	225	96	129	Native population	Ankara	Turkish
14	Gümüş Sekerci, 2020 [41]	Cross-sectional	Married women aged 15–49	389	389	NA	NA	Hatay	English
15	Kiyak, 2020 [42]	Retrospective cohort study	Women of any age with singleton pregnancies	1556	616	940	Native population	Istanbul	English
16	Bayram Deger, 2020 [43]	Cross-sectional, Cross-sectional	Women who had children aged 6–72 months	762	381	381	Native population	Mardin	English
17	Turkay, 2020 [40]	Retrospective, using hospital data	Syrian and Turkish adolescents who had given birth between 2016 and 2017	255	67	188	Native population	Kocaeli	English
11	Demographic and Health Survey, 2019 [62]	Retrospective questionnaire	Syrian and Turkish women who had given birth 2 years before sampling	3253	2168	1085	Native population	Whole country	Turkish
18	Cantürk, 2019 [58]	Retrospective, hospital records	Women who gave birth during 1 year	349	152	197	Native population	Kirsehir	Turkish
19	Sirin, 2019 [59]	Case control, hospital data	Women who gave birth	316	158	158	Native population	Istanbul	Turkish
20	Çelik, 2019 [44]	Retrospective study, hospital data	Newborns	49,413	718	48,504	Native population	Ankara	English
21	Firtana Tuncer, 2019 [45]	Retrospective, using hospital data	Syrian women who had given birth between 2013 and 2016	698	698	NA	NA	Ankara	English
22	Kanmaz, 2019 [46]	Retrospective, hospital data	Pregnant women	17,000	4802	12,198	Yes	Izmir	English
23	Alan Dikmen, 2019 [47]	Cross-sectional, questionnaire	Women aged 18–49 years	555	555	NA	NA	Konya	English
24	WHO report, 2019 [61]	Cross sectional, questionnaire	Women who gave birth in Turkey	624	624	NA	NA	Whole country	English
25	Bayram Deger, 2018 [65]	Cross-sectional, questionnaire	Women of reproductive age	363	363	NA	NA	Mardin	English
26	Ozel, 2018 [49]	Retrospective cohort study using hospital data	Women who gave birth in the Zekai Tahi hospital	1152	576	576	Native population	Ankara	English
27	Güngör, 2018 [50]	Retrospective, using hospital data canturk	Syrian and Turkish women who had given birth between 2016 and 2017	1448	704	744	Native population	Istanbul	English
28	Çift, 2017 [60]	Cross-sectional, questionnaire	Women who gave birth in the training and research hospital	597	297	300	Native population	Bursa	Turkish
29	Simsek, 2017 [53]	Cross-sectional, questionnaire	Married women aged 15–49 years	458	458	NA	NA	Sanliurfa	English
30	Demirci, 2017 [54]	Retrospective, using hospital records	Pregnant women with singleton live births	1090	545	545	Native population	Bursa	English
31	Erenel,2016 [55]	Retrospective cohort study	Women with singleton pregnancies who gave birth in Sisli Hamidiye Etfal training and research hospital	600	300	300	Native population	Istanbul	English
32	Dereli, 2016 ^¥^[63]	Cross-sectional, questionnaire	Pregnant women and women in the postpartum period	400	145	255	Native population	Istanbul	Turkish

*NA* Not applicable

^*﻿^The source of data is the Turkey Demographic and Health Survey collected by the Hacettepe University Institute of Population Studies between October 2018 and February 2019

^¥^Master of Science thesis

### Personal characteristics

#### Age of participants

A total of 16 studies remained for the estimation of the mean and SD of the age of participants [31, 33, 35–39, 42, 50–52, 54, 55, 58–60]. The reason was that 10 investigations either did not report means and standard deviations (SD) [66] for age or provided only medians and ranges [23, 42, 46, 56, 57, 61–63, 67]. Due to the unavailability of raw data, we excluded the mentioned studies from our analysis. Syrian women were on average 2.86 years younger than Turkish women, with a 95% confidence interval ranging from −3.51 to −2.20 (Table 2).

**Table 2:** Personal characteristics of Syrian migrants and Turkish population

Indicator (Qualitative)	Number of studies	Migrant sample size	Native population sample size	Events among migrants	Events among natives	RR 95% CI^¶^	*P* value	I2
Perceived income status (low income) [1–3]	3	4381	16,841	4010	4809	3.23 (1.53 to 6.79)	0.002	99%
Educational status (high school/diploma or higher) [1, 2, 4–9]	8	8211	21,365	741	2372	0.74 (0.44 to 1.25)	0.264	97%
Lack of health insurance [1, 3, 4, 6, 8, 10]	6	4323	5237	883	701	2.03 (0.50 to 8.16)	0.001	96%
Indicator (quantitative)	Number of studies	Migrant population		Native population		MD (95%CI)*	P	I^2^
Weighted mean (SD) of age (years old)	16	24.5(5.8)		27.6(5.9)		−2.86 (−3.51 to −2.20)	0.001	98%
Weighted mean (SD) gravida [3, 9, 11–14]	8	2.29 (1.5)		1.95 (1.29)		0.05 (−0.19 to 0.3)	0.686	97%

Risk ratio (95% confidence interval)

*Mean difference (95% confidence interval)

#### Prevalence of primigravida

Of the 32 studies included, only 5 [33, 34, 49, 55, 68] specified the frequency of first-time pregnant women. Among the studies that compared the frequency of first pregnancies, one [34] did not provide data for the Turkish population. The remaining studies reported this indicator as a mean with SD [39, 49, 50, 54, 60, 63]. We compared means (SD) for migrant and native populations, and found no difference between groups as regards pregnancy numbers; the data are summarized in Table 2.

#### Perceived income status

Three studies [35, 62, 63] comprising 4,809 Syrian migrants and 16,841 individuals from the native population provided data on perceived income. None of the studies reported exact income figures, but only perceived income levels. The proportion of poor individuals among migrants was approximately three-fold higher than the numbers in the native population, with a risk ratio (RR) of 3.23 and a 95% confidence interval (CI) of 1.53 to 6.79 (Table 2).

#### Educational status

Eight studies [31, 33, 35, 42, 49, 56, 62, 64] comprising a total population of 8,211 Syrian migrants and 21,365 individuals from the native population provided data on educational status. There were no significant differences between groups regarding the prevalence of women with a high school diploma or higher education (Table 2).

##### Lack of health insurance

Six studies [31, 47, 56, 62–64] comprising a total population of 4323 Syrian migrants and 5237 individuals from the native population reported on health insurance status. There were no significant differences between the two groups in regard of health insurance (Table 2).

#### Maternal health outcomes

Seventeen studies assessed maternal health outcomes including anemia, adolescent pregnancy, antenatal care (ANC), cesarean section, gestational diabetes (GDM), home birth preeclampsia/eclampsia, iron supplementation during pregnancy, and unwanted pregnancy. Fifteen studies investigated neonatal health outcomes including the Apgar score, fetal anomalies and macrosomia, stillbirth, low birth weight (LBW), neonatal intensive care (NICU), preterm birth and stillbirth.

#### Anemia and iron supplement during pregnancy

**Fig. 2:**
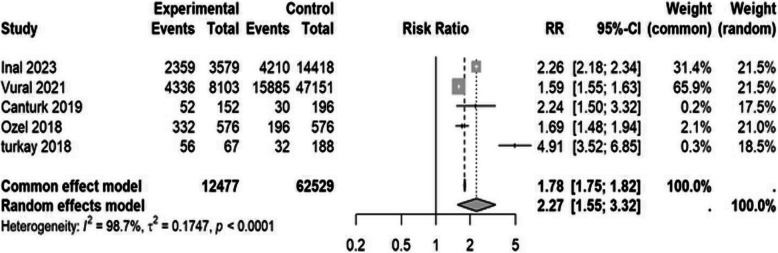
Forest plot of anemia by migration status. The experimental group consisted of Syrian migrants while the control group was the native Turkish population. RR denotes the risk ratio and CI the confidence interval

Five studies [35, 38, 40, 49, 58] reported on anemia during the third trimester of pregnancy. The RR for anemia between migrants and the native population was 2.27 (95% CI: 1.55 to 3.32), indicating that migrants are more than twice as likely to experience anemia compared to their native counterparts (Table 3 and Fig. 2).

**Table 3:** Maternal and newborn health indicators in Syrian migrants and the Turkish population

Maternal health outcomes								
Outcome	Number of studies	Migrant sample size	Native population sample size	Events among migrants	Events among natives	RR 95% CI	*P* value	I2
Anemia [2, 9, 15–17]	5	12,477	62,529	7135	20,353	2.27 (1.55 to 3.32)	0.001	98%
Adolescent pregnancy [1–3, 5–7, 9, 15, 16, 18–22]	14	28,311	264,425	4548	11,341	3.78 (3.06 to 4.88)	0.001	86%
ANC4* [1, 15, 19, 20, 23, 24]	6	3618	15,401	6878	13,466	0.39 (0.26 to 0.58)	0.001	96%
Cesarean section [1–4, 7, 9, 12–16, 18, 22–26]	17	33,794	232,337	12,146	48,046	0.79 (0.49 to 1.38)	0.432	96%
GDM¥ [2, 3, 12, 13, 22–24, 26]	8	10,620	36,456	157	1325	0.44 (0.21 to 0.90)	0.025	80%
Home birth [1, 4, 19, 27]	3	3544	5304	208	74	3.68 (2.53 to 5. 27)	0.001	48%
Preeclampsia/Eclampsia [2, 3, 7, 9, 12, 13, 16, 20, 22–24, 26]	12	20,215	85,383	531	4115	0.68 (0.40 to 1.15)	0.155	92%
Received iron supplement during pregnancy [9, 13, 24]	3	6082	13,518	2350	10,405	0.69 (0.46 to 0.96)	0.030	97%
Unwanted pregnancy [4, 28]	2	2027	2988	216	484	0.70 (0.38 to 1.06)	0.333	58.7%
Neonatal Health Outcome								
5-minuteApgar score < 7 [2, 7, 9, 15, 16, 20, 24]	7	17,906	74,149	372	1117	0.99 (0.28 to 3.50)	0.978	96%
Fetal anomalies [2, 7, 9, 13, 16, 20, 28]	7	8950	64,362	73	276	1.41(0.78 to 2.57)	0.249	78%
Fetal macrosomia [7, 9, 14, 16, 18, 23, 24]	7	25,578	201,346	777	11,141	0.54 (0.50 to 0.58)	0.001	10%
LBW ± [2, 7, 14–16, 18, 20–24, 26]	12	30,404	272,300	3411	29,314	0.93 (0.83 to 1.04)	0.232	79%
NICU admission [2, 9, 13, 16, 21, 22, 24, 26, 29]	8	18,942	130,099	1870	12,932	1.4264 (0.68 to 2.95)	0.341	99%
Preterm birth [1, 4, 6, 7, 9, 14, 16, 18, 22–24, 26, 28, 30]	15	28,925	263,530	4996	36,616	1.21 (0.94 to 1.61)	0.123	91%
Stillbirth [2, 5, 7, 9, 14, 16, 18, 20, 23, 24, 28]	11	29,948	218,127	378	2950	0.99 (0.65 to 1.45)	0.962	80%

Risk ratio (95% confidence interval)

^*^Antenatal care visits at least four times

¥ Gestational diabetes

± Low birth weight

**Fig. 3:**
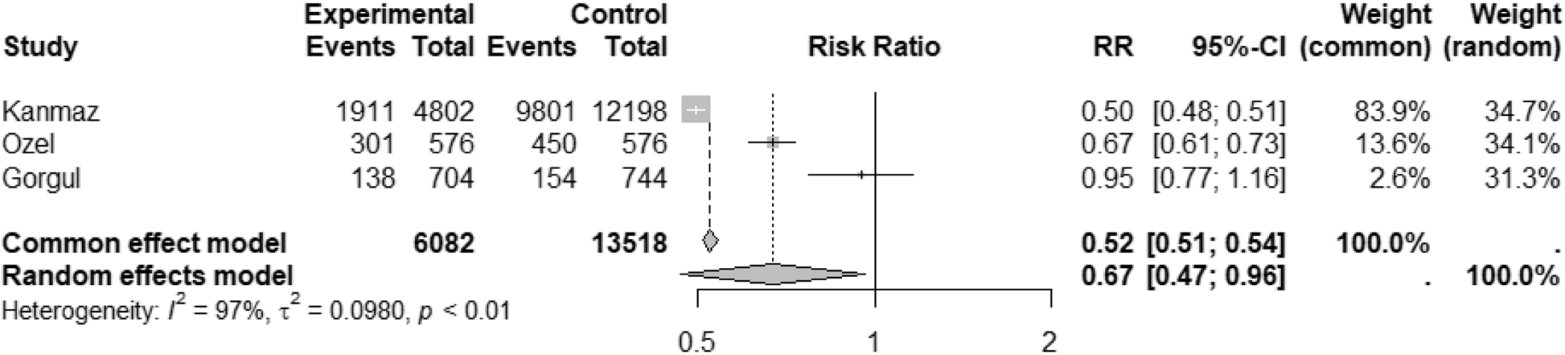
Forest plot of received iron supplement during pregnancy by migration status. The experimental group consisted of Syrian migrants while the control group was the native Turkish population. RR denotes the risk ratio and CI the confidence interval

The results of 3 studies [46, 49, 50] showed that Syrian migrants had less access to iron supplementation throughout pregnancy (RR 0.69, 95% CI 0.46 to 0.96) (Tables 3 and 4. A sensitivity analysis of 4 studies [32, 46, 49, 50] revealed similar results (RR 0.69, 95% CI 0.48 to 0. 99) (Table and Fig. 3).

#### Adolescent pregnancy

**Fig. 4:**
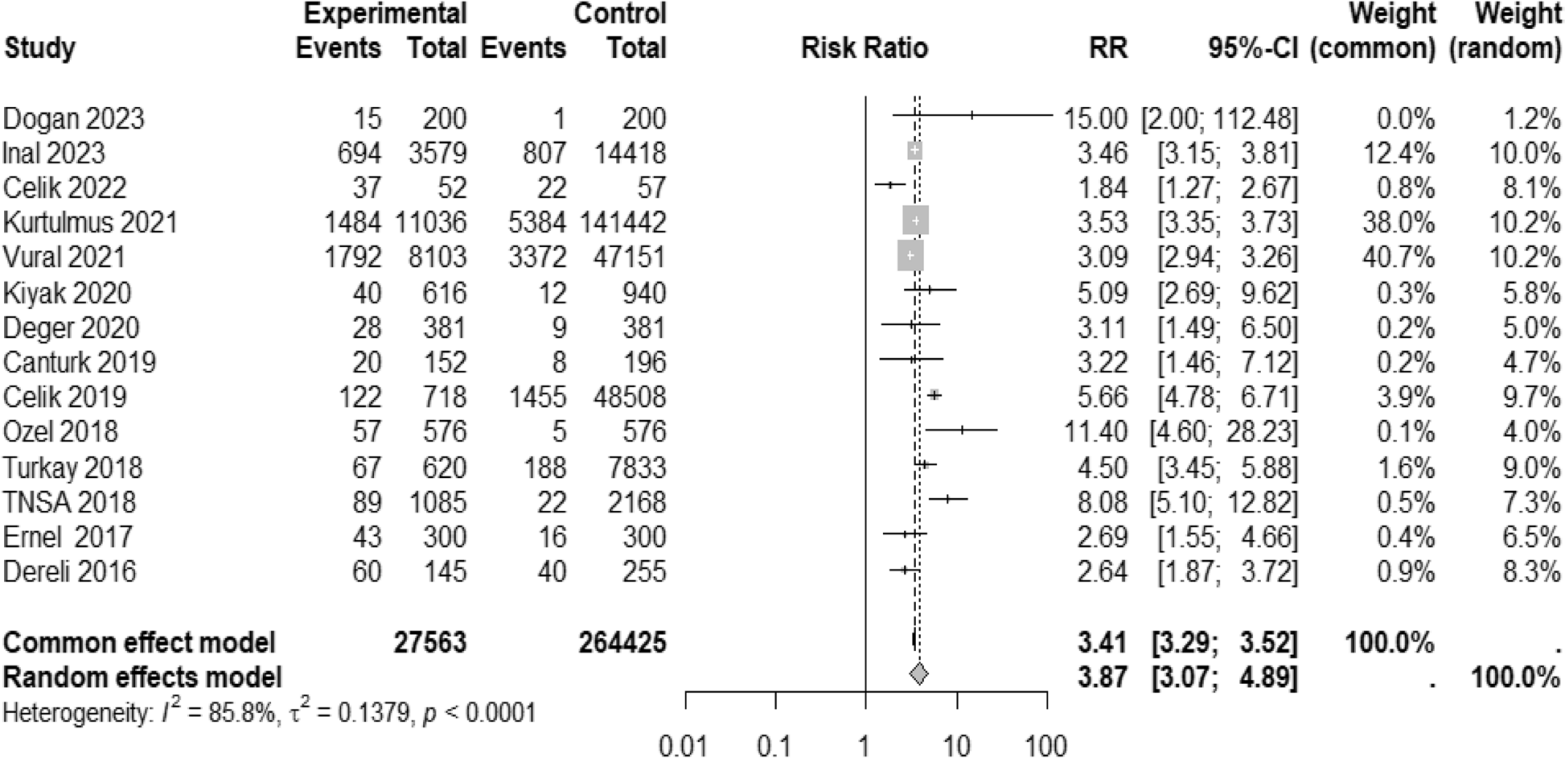
Forest plot of adolescent pregnancy prevalence by migration status. The experimental group consisted of Syrian migrants while the control group was the native Turkish population. RR denotes the risk ratio and CI the confidence interval

Fourteen studies [33, 35, 36, 38, 42–44, 49, 52, 55, 56, 58, 62, 63] reported on this outcome. The risk of adolescent pregnancy among Syrian migrants was approximately fourfold higher than the risk in the native Turkish population (RR 3.87, 95% CI 3.06 to 4.88) (Table 3 and Fig. 4). A sensitivity analysis of sixteen studies [33–36, 38, 42–44, 49, 52, 53, 55, 56, 58, 62, 63] showed similar results (Table 4).

**Table 4:** Maternal and newborn health indicators in Syrian migrants and the Turkish population (sensitivity analysis)

Indicator	Number of studies	Migrant sample size	Native population sample size	Events among migrants	Events among natives	RR lower upper	*P* value	I^2^
Maternal Health Outcomes								
Adolescent pregnancy [1–3, 5–7, 9, 15, 16, 18–22, 31, 32]	16	28,311	264,465	4693	11,341	3.87 (3.06 to 4.88)	0.001	85.8%
ANC4* [1, 15, 19, 20, 23, 24, 33, 34]	9	8380	15j401	4094	13j466	0.42 (0.29 to 0.60)	0.001	98%
Cesarean section [1–4, 7, 9, 12–16, 18, 22–26, 34]	18	35,492	232,337	12,405	48,046	0.88 (0.56 to 1.38)	0.586	99%
Contraception prevalence [4, 8, 10]	3	2902	2755	1019	1353	0.7 (0.27 to 1.83)	0.721	22.8%
GDM¥ [2, 3, 12, 13, 22–24, 26, 29]	9	13,486	36,456	188	1325	0. 37 (0.17 to 0.79)	0.010	91%
Received iron supplements during pregnancy [9, 13, 24, 29]	4	8948	104,405	4659	10j405	0.69 (0.48 to 0.99)	0.041	92
Preeclampsia/Eclampsia [2, 3, 7, 9, 12, 13, 16, 20, 22–24, 26, 29, 34]	14	23,779	85,383	583	4115	0.56 (0.32 to 0.98)	0.001	82%
Neonatal Health Outcome								
Fetal anomalies [2, 7, 9, 13, 16, 20, 28, 29]	9	11,816	64,362	162	276	1.19 (0.63 to 2.26)	0.582	79%
Fetal macrosomia [7, 9, 14, 16, 18, 23, 24, 29]	8	28,444	201,346	866	11,141	0.54 (0.49 to 0.58)	0.001	10%
NICU admission [2, 9, 13, 16, 21, 22, 24, 26, 29, 34]	10	22,512	130,099	2089	12,932	1.17 (0.57 to 2.38)	0.655	98%
Preterm birth [1, 4, 6, 7, 9, 14, 16, 18, 22–24, 26, 28, 30, 34]	16	29,623	263,530	5130	36,616	1.21 (0.93 to 1.58)	0.138	91%
Stillbirth [2, 5, 7, 9, 14, 16, 18, 20, 23, 24, 28, 29, 31]	12	32,814	218,127	396	2950	0.99 (0.65 to 1.48)	0.962	80%

^*^Antenatal care visits at least four times

¥ Gestational diabetes

#### ANC

**Fig. 5:**
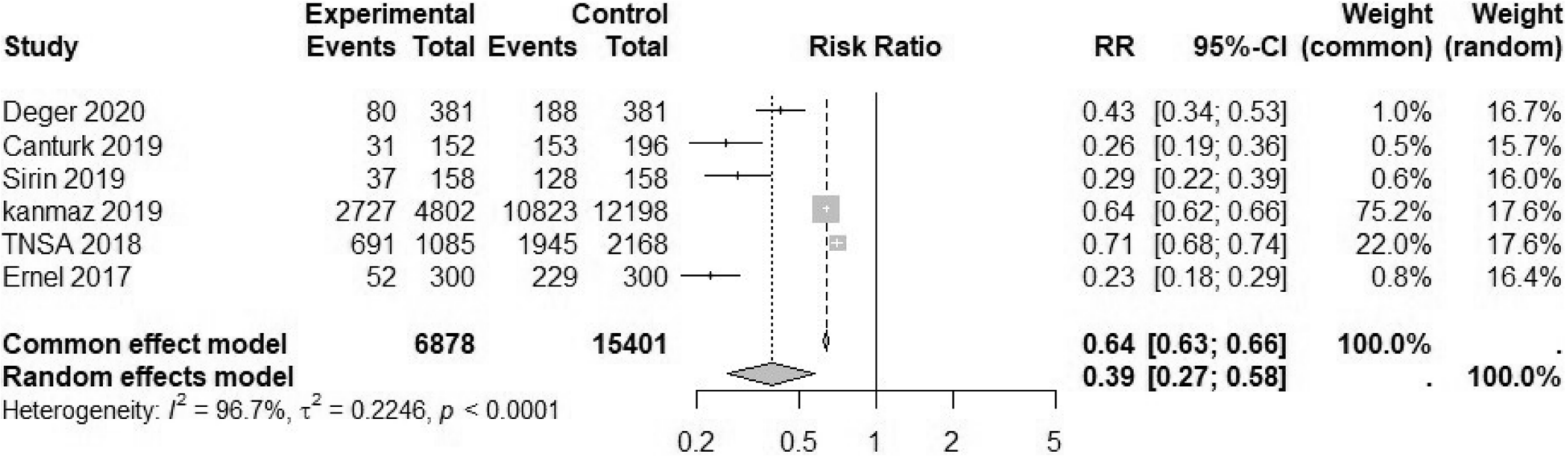
Forest plot of antenatal care (at least 4 times) by migration status. The experimental group consisted of Syrian migrants while the control group was the native Turkish population. RR denotes the risk ratio and CI the confidence interval

Six studies [43, 46, 55, 58, 59, 62] investigated ANC. For the purpose of this meta-analysis, the minimum threshold for ANC was defined as four visits during pregnancy. The results indicated that migrants had significantly less access to at least four ANC visits compared to the native population. (RR 0.39, 95% CI 0.29 to 0.58) (Table 3 and Fig. 5). A sensitivity analysis of 8 studies [43, 45, 46, 55, 58, 59, 62, 65] showed similar results (Table 4).

#### Cesarean section rate

The results of 17 [31, 35, 36, 38, 39, 42, 46, 49–52, 54, 58, 59, 62, 63, 67] studies that assessed cesarean section rates among migrants and the native Turkish population indicated no significant difference between the two groups (Table 3). A sensitivity analysis of 18 studies [31, 35, 36, 38, 39, 42, 45, 46, 49–52, 54, 58, 59, 62, 63, 67] also did not reveal any difference between groups (Table 4).

#### Contraception use and unwanted pregnancy

We assessed modern contraception methods, including oral contraceptives, condoms, intrauterine devices (IUDs), and injectable contraception. Three studies [31, 47, 64] reported on the use of modern contraceptive methods and revealed no significant difference between the two groups (Table 4).

Only two studies [31, 37] examined unwanted pregnancy, and the analysis indicated no difference between groups (RR 0.70, 95% CI 0.38 to 1.06) (Table 3).

#### GDM and fetal macrosomia

**Fig. 6:**
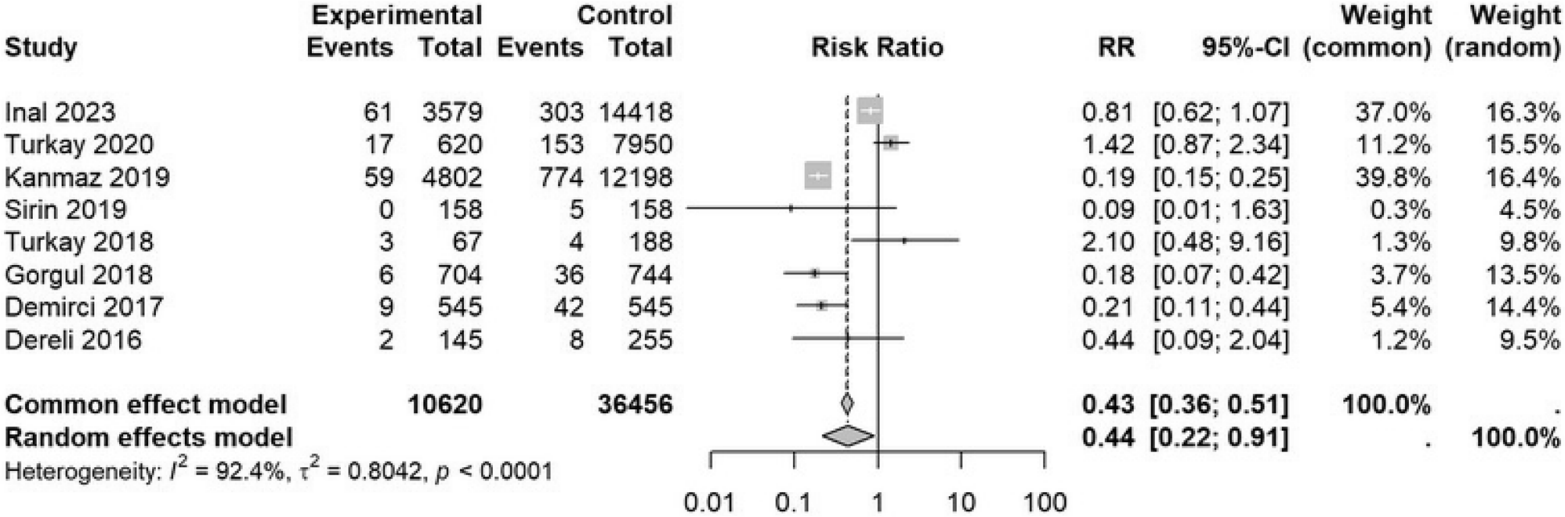
Forest plot of gestational diabetes by migration status. The experimental group consisted of Syrian migrants while the control group was the native Turkish population. RR denotes the risk ratio and CI the confidence interval

Eight studies [35, 46, 50–52, 54, 59, 63] examined gestational diabetes mellitus (GDM). Being a migrant had a protective effect in regard of GDM (RR 0.44, 95% CI 0.21 to 0.9) (Table 3). A sensitivity analysis showed similar results (Table 3 and Fig. 6). A further sensitivity analysis of 9 studies [32, 35, 46, 50–52, 54, 59, 63] revealed similar results (RR 0.37, 95% CI 0.17 to 0. 79) (Table 4).

Being a migrant was a protective factor in regard of fetal macrosomia in 7 studies [36, 38, 39, 42, 46, 49, 59], (RR 0.54, 95% CI 0.50 to 0. 58) (Table 3) as well as in a sensitivity analysis of eight studies [32, 36, 38, 39, 42, 46, 49, 59] (RR 0.54, 95% CI 0.49 to 0. 58) (Table 4).

#### Home birth

**Fig. 7:**
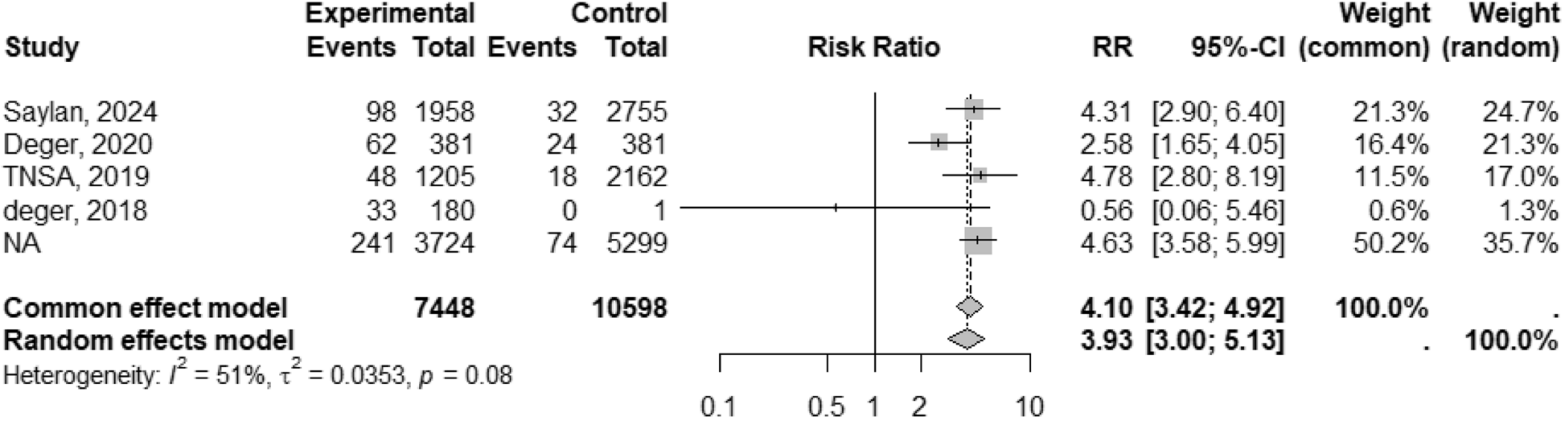
Forest plot of home birth by migration status. The experimental group consisted of Syrian migrants while the control group was the Turkish native population. RR denotes the risk ratio and CI the confidence interval

Four studies [31, 43, 48, 62] reported on the prevalence of home births. Syrian migrants were nearly four times more likely to have home births (RR 3.68, 95% CI 2.53 to 5.27) (Table 3 and Fig. 7).

#### Preeclampsia/eclampsia

**Fig. 8:**
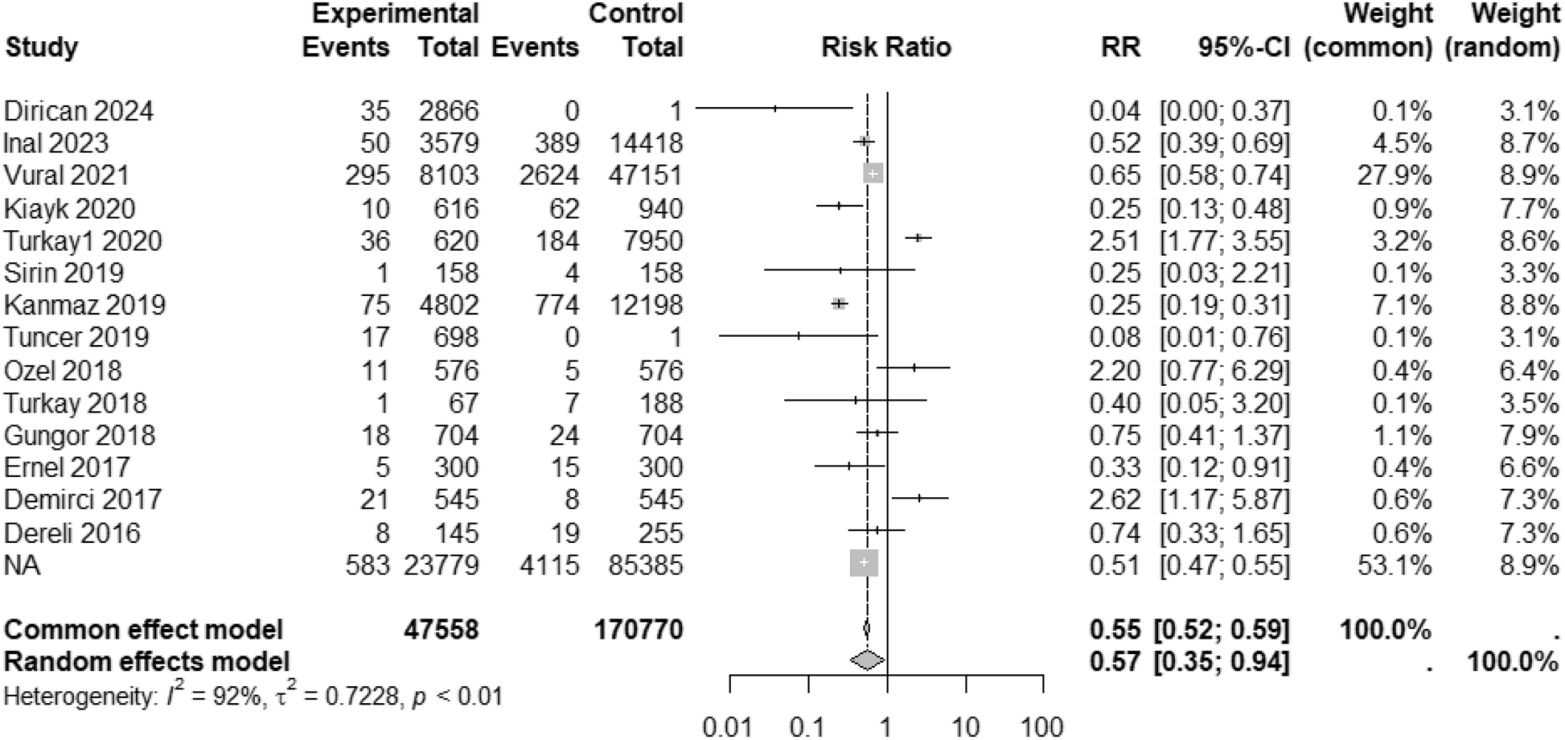
Forest plot of preeclampsia/eclampsia by migration status. The experimental group consisted of Syrian migrants while the control group was the native Turkish population. RR denotes the risk ratio and CI the confidence interval

While an analysis of 12 studies [35, 38, 42, 46, 49–52, 54, 55, 59, 63] did not reveal a significant difference between groups in regard of preeclampsia/eclampsia (Table 3), a sensitivity analysis of 14 studies [32, 35, 38, 42, 45, 46, 49–52, 54, 55, 59, 63] indicated that being a migrant may serve as a protective factor against preeclampsia/eclampsia (RR 0.56, 95% CI 0.32 to 0.98) (Table 4 and Fig. 8).

### Neonatal health outcome

#### 5-min Apgar score

The results of seven studies [35, 38, 42, 46, 49, 55, 58] revealed no difference between migrant and native newborns in terms of a 5-min Apgar score below 7 (Table 3).

#### Fetal anomalies

Fetal anomalies were assessed in seven studies [35, 37, 38, 42, 49, 50, 55] and revealed no difference between the migrant and native populations (Table 3). A sensitivity analysis of eight studies [32, 35, 37, 38, 42, 49, 50, 55] yielded the same result (Table 4).

##### Low birth weight

Twelve [35, 36, 38, 39, 42, 44, 46, 51, 52, 55, 58, 59] studies reported no difference between the two groups in terms of LBW newborns (Table 3).

#### Admission

The results of 8 [32, 35, 38, 44, 46, 49–52] studies that assessed the NICU admission rate of newborns immediately after birth among migrants and the Turkish native population indicated no significant difference between the two groups (Table 3). A sensitivity analysis of 10 studies [32, 35, 38, 44–46, 49–52] also revealed no difference between groups (Table 4).

#### Preterm birth

Fifteen studies [31, 36–39, 42, 46, 49, 51, 52, 56, 57, 59, 62] addressed preterm births among migrants and the Turkish native population, and reported no significant difference between groups (Table 3). Additionally, a sensitivity analysis based on 16 studies [31, 36–39, 42, 45, 46, 49, 51, 52, 56, 57, 59, 62] yielded no differences between groups (Table 4).

#### Stillbirth

Eleven studies [33, 35–39, 42, 46, 49, 55, 59] investigated stillbirth rates among migrants and the native Turkish population, and reported no significant differences between groups (Table 3). A sensitivity analysis of 12 studies [32–39, 42, 46, 49, 55, 59] corroborated these results, showing no differences between the groups (Table 4).

#### Quality assessment

Eighteen studies [31, 32, 35, 36, 38, 42, 43, 45, 46, 51–54, 56, 57, 63, 64, 69] were of high quality, 7 [47, 49, 50, 55, 58–60] of moderate quality, and 5 [33, 34, 39, 44, 65] of poor quality; the results are summarized in Table 5.

**Table 5:** Quality assessment of the studies included in the meta-analysis

Study	Were the criteria for inclusion in the sample clearly defined?	Were the study subjects and the setting described in detail?	Was the exposure measured in a valid and reliable way?	Were objective standard criteria used for measurement of the condition?	Were confounding factors identified?	Were strategies to deal with confounding factors stated?	Were the outcomes measured in a valid and reliable way?	Was appropriate statistical analysis performed?	Score/Quality
Saylan, 2024 [31]	1	1	1	1	0	0	1	1	6/High
Dirican, 2024 [32]	1	1	1	1	1	1	1	1	8/High
Dogan, 2023 [33]	1	1	1	1	0	0	0	0	4/Low
Dogan, 2023 [34]	1	1	1	1	0	0	0	0	4/Low
Inal, 2023 [35]	1	1	1	1	0	0	1	1	6/High
Çelik, 2022 [56]	1	1	1	1	0	0	1	1	6/High
Karaca Kurtulmus, 2021 [36]	1	1	1	1	0	0	1	1	6/High
Sayili, 2021 [37]	1	1	1	1	1	1	1	1	8/High
Vural, 2021 [38]	1	1	1	1	1	1	1	1	8/High
Kanawati,2021 [39]	1	0	1	0	0	0	1	1	4/Low
Turkay, 2020 [40]	1	1	1	1	0	0	1	1	6/High
Okman, 2020 [57]	1	1	1	1	0	0	1	1	6/High
Gümüş Sekerci, 2020 [41]	1	1	1	1	0	0	1	1	6/High
Kiyak, 2020 [42]	1	1	1	1	0	0	1	1	6/High
Bayram Deger, 2020 [43]	1	1	1	1	0	1	0	1	6/High
Turkay, 2020 [40]	1	1	1	1	1	1	1	1	8/High
Cantürk, 2019 [58]	1	1	1	1	0	0	0	1	5/Moderate
Sirin, 2019 [59]	1	1	1	1	0	0	0	1	5/Moderate
Çelik, 2019 [44]	0	1	1	1	0	0	1	0	4/low
Firtana Tuncer, 2019 [45]	1	1	1	1	0	0	1	1	6/High
Kanmaz, 2019 [46]	1	1	1	1	1	1	1	1	8/High
Alan Dikmen, 2019 [47]	1	1	1	1	0	0	1	0	5/Moderate
Bayram Deger, 2018 [65]	1	1	1	1	0	0	0	0	4/low
Ozel, 2018 [49]	1	1	1	1	0	0	1	0	5/Moderate
Güngör, 2018 [50]	1	1	1	1	0	0	1	0	5/Moderate
Çift, 2017 [60]	1	1	1	1	0	0	0	1	5/Moderate
Simsek, 2017 [53]	1	1	1	1	1	1	1	1	8/High
Demirci, 2017 [54]	1	1	1	1	1	1	1	1	8/High
Erenel, 2016 [55]	1	1	0	1	0	0	1	1	5/Moderate
Dereli, 2016 [63]	1	1	1	1	0	0	1	1	6/High

## Discussion

Of the 15 assessed indicators, 8 demonstrated significant inequalities between the migrant and native populations, including anemia, receipt of iron supplementation during pregnancy, antenatal care coverage, the prevalence of gestational diabetes mellitus (GDM), preeclampsia/eclampsia, macrosomia, the prevalence of home births, and unwanted pregnancies. Additionally, the results of the present meta-analysis indicated that the migrant population was younger and had a higher proportion of individuals with low incomes and a lack of health insurance compared to the native population.

The present meta-analysis revealed a significant difference in ANC utilization between migrants and the native population. Turkish individuals accessed antenatal care roughly twice as often as migrants, based on a benchmark of at least four visits during pregnancy [70]. Notably, two other outcomes related to ANC—receiving iron supplementation during pregnancy and anemia in the third trimester—also showed significant differences between the two groups. The calculated risk ratio for anemia among migrants was (RR: 2.27), highlighting the greater vulnerability of migrant women to this condition. Additionally, the risk ratio for home births among migrants was 3.68, indicating that they are significantly more likely to have home births compared to the native population.

Generally, migrant and refugee women utilize antenatal care services less frequently than their counterparts in host countries, despite the associated health risks. Research in the United States showed that refugee and migrant women are more prone to postponing their antenatal care appointments than local women [69, 71, 72]. While maternal services are free of charge for Syrian migrants at least in their registered region of the country, it appears that other factors including language barriers, cultural differences, transportation difficulties, financial constraints, and concerns about discrimination [71, 73] may lead to a lower usage of ANC among these women. Our analysis revealed that the proportions of low-income persons and those with no health insurance were higher in the migrant population. These two factors explain the more numerous home births in the migrant population.

Interestingly, being a migrant was a protective factor in regard of GDM, hypertensive disorders including preeclampsia/eclampsia, and fetal macrosomia. Age is a well established risk factor for both GDM and preeclampsia [74, 75]. An investigation conducted by Shuang et al. demonstrated that women aged 35 years and older have an odds ratio of 1.15 (95% CI: 1.05–1.26) for developing GDM [76]. A comparative study by Li et al. involving 48 women with gestational diabetes and 202 non-diabetic women reported that the average age in the GDM group was higher than that in the non-GDM group. Notably, the study revealed a 12.5% increase in the risk of GDM with each additional year of maternal age [77]. Investigations conducted by Schummers [78] and Li [79] supported these findings, indicating a linear increase in the risk of GDM with advancing maternal age, particularly in women over the age of 24 years. Harakow et al., in their meta-analysis, found that migrant women are less affected by hypertensive disorders during pregnancy, including preeclampsia [80]. The lower incidence of GDM and hypertensive disorders among Syrian migrants may be linked to their age and multiparity, which aligns with the"migrant health effect."This hypothesis suggests that many women who migrate to a different country tend to be healthier and younger than those who remain in their home country. As a result, they bring certain health advantages with them compared to the average individual in their country of origin [81].

The limitations of this systematic review are worthy of note. One significant limitation is the reliance on wealth index assessments from a limited number of studies. Specifically, many studies included in this analysis evaluated wealth using a single subjective question and did not permit a comprehensive assessment of wealth. This approach may not accurately capture the true socio-economic status of individuals.

## Conclusion

Our findings indicate that the migrant population in Turkey may face challenges in accessing maternal care. One major factor is that migration is accompanied with discrimination for all refugees, but particularly for the fragile group of refugee pregnant women and their unborn children. This may be due to language barriers or financial issues, as the prevalence of low-income individuals is higher among migrants than in the native population. Migrants have higher odds of adolescent pregnancy, which highlights the issue of poverty in this group. One notable outcome of this meta-analysis was that being a migrant could be a protective factor against systemic diseases, including GDM and preeclampsia/eclampsia. This may be related to the younger age of migrant mothers compared to the native population. Addressing these disparities is crucial for improving maternal and newborn health outcomes among migrants. Failing to do so will impair health in the long term and jeopardize the elimination of health inequities.

## Data Availability

No datasets were generated or analysed during the current study.
